# Infections and Their Outcomes in Cancer Patients With and Without Neutropenia: A Single-Center Experience

**DOI:** 10.7759/cureus.51983

**Published:** 2024-01-09

**Authors:** Renu Mishra, Arpan Patel, Ankit Agarwal, Dilshano Thaiyam, Shehdil Thaiyam

**Affiliations:** 1 Department of Medical Oncology, Geetanjali Medical College & Hospital, Udaipur, IND

**Keywords:** hematological malignancy, solid malignacy, rti, soft tissue infection, uti, bsi, non-neutropenic episode, neutropenia, infections, cancer

## Abstract

Background

Patients with cancer are at a high risk of developing infections due to immunosuppression resulting from cancer treatment. Infections may occur both during neutropenic and non-neutropenic episodes and negatively impact outcomes both in terms of hospital stay and mortality. In this study, we aimed to determine the infection types, microbiological picture of infections, their outcomes, and associated factors in cancer patients during neutropenic and non-neutropenic episodes.

Methods

This is a retrospective cross-sectional study conducted at the Department of Medical Oncology, Geetanjali Medical College & Hospital, a tertiary care hospital in northern India. A total of 82 cancer patients with infections between August 2021 and July 2022 were included in this study.

Results

A total of 82 patients had 96 episodes of infections. Out of 82 patients, 24 (29.3%) had hematological malignancies, and 58 (70.7%) had solid malignancies. The majority of episodes (n = 60; 62.5%) were seen in patients with solid malignancies, and the rest (n = 36; 37.5%) of them were seen in patients with hematological malignancies. Among all the episodes of infection, 28 (29.2%) were encountered during neutropenic episodes, while the rest (n = 68; 70.8%) of the incidences were encountered during non-neutropenic episodes. Out of 28 neutropenic episodes of infection, the majority (n = 23; 82.1%) occurred in patients with hematological malignancies. An absolute neutrophil count (ANC) of <500 cells/mm^3^ (severe neutropenia) was present in 26 (92.8%) patients in the neutropenic group. There was no major difference in causative microbiology among both groups. Gram-negative organisms were the predominant pathogens in both groups. *Escherichia coli* was the most commonly isolated, followed by *Klebsiella pneumoniae *and *Candida *spp. The mortality rate was 12.5%, with a significantly higher mortality in the neutropenic group (odds ratio (OR) 3.4, 95% confidence interval (CI) 1.178-9.813; p = 0.042). Neutropenic patients also had a longer median length of stay (LOS, 10 days) as compared to non-neutropenic patients (seven days).

Conclusion

This study revealed a high frequency of neutropenia in patients with hematological malignancies. Gram-negative pathogens were the major causative organisms of infection in both patient groups. *E. coli* infection rates were high in both groups. Neutropenic patients had significantly higher mortality rates and a longer LOS compared to non neutropenic patients.

## Introduction

Infections in cancer patients are still one of the major factors contributing to added hospital stays, cancer-related deaths, and the cost of treatment [1]. Chemotherapy-induced neutropenia occurs much faster compared to anemia and thrombocytopenia, attributed to the significantly shorter half-lives of granulocytes as compared to erythrocytes and thrombocytes [2]. Fever was ranked third and fourth in two studies assessing the most frequent reasons cancer patients visit the emergency department, respectively [3,4]. The common causes of fever in cancer patients are infections, fever of unknown origin, drugs, metastasis, transfusion reactions, and other miscellaneous causes. However, infections are the most common cause of fever in cancer patients [3,5]. 

Infectious episodes among cancer patients are not just limited to patients who develop neutropenia during treatment but occur in non-neutropenic patients as well. Studies on infections in cancer patients with neutropenia receive a lot of attention; however, infections in patients who are not neutropenic also need to be studied more closely. An analysis of emergency room visits by cancer patients with non-neutropenic fever revealed that a significant percentage of these patients (83%) required inpatient admission, of which 76% were diagnosed with an infection [6].

In day-to-day clinical practice and as per previous studies, patients with cancer also suffer from infections that are only clinically diagnosed or documented without the isolation of a pathogen. One such study showed only clinically documented infections without isolation of a pathogen in 8.7% of neutropenic episodes and 10.3% of non-neutropenic episodes [7], and another study showed the same among 21% of patients with febrile neutropenia [8].

Most of the currently published literature focuses on studies done on infections in hematological malignancies, neutropenic patients, and blood stream infections (BSIs). There are fewer studies done on the comparative characteristics of infection between neutropenic and non-neutropenic cancer patients, as well as outcomes in terms of length of hospital stay (LOS) and mortality. In the current study, we aim to describe the characteristics of infection in cancer patients with hematological and solid malignancy during neutropenic and non-neutropenic episodes. In this study, we also aimed to explore the factors associated with outcomes. We also describe infections at sites other than BSIs along with their microbiological profiles. Our findings will enhance understanding and guide interventions for improved outcomes in the realm of cancer-associated infections.

## Materials and methods

A retrospective cross-sectional study was conducted at the Department of Medical Oncology, Geetanjali Medical College & Hospital, Udaipur, Rajasthan (India). We obtained patient data between July 2021 and August 2022 from hospital medical records. We obtained the medical records of patients with both solid and hematological malignancies. After analysis of the patient records, we included patients who had the following findings: (I) the clinical manifestations of infection and (II) positive culture results for the presence of pathogenic organisms from an appropriate sample (blood, urine, bronchial aspirate, sputum, and pus). Patients with only a clinical diagnosis of infection or hospital-acquired infection were not included. Two patients in our study had two site infections (BSI and urinary tract infection (UTI)) at the presentation; both of them were considered to have had one episode of infection each. Normal commensals and contaminants were included only if they were isolated from a repeated sample. Outcomes measured were median LOS and discharge from the hospital or mortality. The LOS was considered to be the duration between admission and hospital discharge. Neutropenia was defined as an absolute neutrophil count (ANC) of <1500 cells/mm^3^ [9]. Neutropenia was further categorized as mild (ANC 1000-1500 cells/mm^3^), moderate (ANC 500-1000 cells/mm^3^), severe (ANC <500 cells/mm^3^) [9], or profound neutropenia (ANC <100 cells/mm^3^) [10].

The study was approved by the Human Research Ethics Committee (HREC) Geetanjali Medical College and Hospital, Udaipur (Certificate No.: GU/HREC/EC/2022/2024 and GU/HREC/EC/2022/2025)

IBM SPSS Statistics for Windows, version 27.0 (released 2020, IBM Corp., Armonk, NY) and Microsoft Excel (Microsoft Corp., 2021) were used to pool all the data and analyze it. We used percentages and numbers for descriptive analysis of categorical variables, and mean, standard deviation, and median were used for continuous variables. The chi-square test, Fisher's exact test, independent sample T-test, and Mann-Whitney U test were used wherever applicable. A p-value of <0.05 was assumed to be significant, and a confidence interval (CI) of 95% was considered.

## Results

During the study period, a total of 82 patients (41 male and 41 female) with cancer, including those with solid malignancies and hematological malignancies, tested positive for infections through microbiological testing (Table 1). Out of the 82 patients, 24 were found to have hematological malignancies and 58 had solid malignancies (Table 2). These 82 patients had a total of 96 incidences of infection (Table 3). A total of 98 unique samples tested positive for infectious organisms. Patients with solid malignancy had 60 (62.5%) episodes of infection, and those with hematological malignancy had 36 (37.5%) episodes of infection. Among all the episodes, 28 incidences (29.2%) and 68 (70.8%) incidences of infection were encountered during neutropenic and non-neutropenic episodes, respectively. Neutropenic patients had a lower mean age than non-neutropenic patients (p < 0.05). Out of 28 neutropenic patients, 27 had severe neutropenia (ANC <500 cells/mm^3^), and only one patient had moderate neutropenia (ANC 500-1000 cells/mm^3^). There were zero patients with mild neutropenia (ANC 1000-1500 cells/mm^3^). Out of 27 patients with severe neutropenia, nine (33.3%) were found to have profound neutropenia (ANC <100 cells/mm^3^). The frequency of neutropenic episodes was higher in patients with hematological malignancies (p < 0.05).

**Table 1:** Demographic distribution of the patients

	Neutropenic episode	Non-neutropenic episode	Total	p-value
Gender				
Male	16	35	96	0.613
Female	12	33		
Age range				
0-15	10	5	96	<0.001
16-30	6	5		
31-45	4	12		
46-60	6	27		
61-75	1	18		
>75	1	1		
Mean age (SD)	28.2(22.5)	48.7(19)	42.7(22)	<0.001
Median age	19	53	48.5	<0.001
Cancer type				
Solid	5	55	96	<0.001
Hematological	23	13		

**Table 2:** Type of malignancies Other gastrointestinal malignancies include gastric, gall bladder, liver, pancreas, and rectum.

Cancer type	Neutropenic	Non-neutropenic
Acute lymphocytic leukemia (ALL)	11	10
acute myelogenous leukemia (AML)	7	0
Chronic myeloid leukemia (CML)	1	1
Multiple myeloma	2	0
Non-Hodgkin's lymphoma (NHL)	2	2
Lung	0	12
Breast	1	1
Esophagus	0	6
Oral	1	6
Other gastrointestinal malignancies	0	8
Prostate	0	3
Uterus	1	1
Ovary	1	2
Cervix	0	7
Nasopharynx	0	2
Urothelial	0	4
Mesothelioma	0	1
Bone	1	1
Liposarcoma	0	1

**Table 3:** Infection type UTI: urinary tract infection; BSI: blood stream infection; RTI: respiratory tract infection

Infection type	Neutropenic episode	Non-neutropenic episode	Total
UTI	09	25	34
BSI	15	09	24
RTI	3	23	26
BSI and UTI	1	1	02
Soft tissue	0	10	10
Total	28	68	96

Gram-negative isolates constituted the majority (n = 75; 76.5%), while gram-positive isolates accounted for a smaller proportion (n = 12; 12.2%), and fungal organisms were identified in a lesser number (n = 11; 11.2%). *Escherichia* *coli* was the most common (n = 40) organism isolated across all the samples. Other major organisms isolated were *Klebsiella* *pneumoniae* (n = 20), *Candida* spp. (n = 11), *Pseudomonas* *aeruginosa* (n = 7), *Staphylococcus* spp. (n = 7), and *Enterococcus* spp. (n = 5). One patient had a BSI with *Ralstonia mannitolytica*, a rare cause of BSI. Out of 11 fungal isolates, seven were *C. albicans*, and only four were non-*C. albicans* species. All the patients with BSI in the neutropenic group had severe neutropenia (ANC <500 cells/mm^3^) (see Table 4).

**Table 4:** Summary of infectious agents isolated from various sites of all the patients involved in this study

Sample types	Organism isolated	Neutropenic episode	Non-neutropenic episode	Counts
Blood	Candida albicans	1	0	1
	Escherichia coli	6	3	9
	*Enterococcus *spp*. *	0	1	1
	Klebsiella pneumoniae	5	1	6
	Pseudomonas aeruginosa	0	1	1
	Ralstonia mannitolitytica	1	0	1
	Sphingomonas Paucimobilis	0	1	1
	Staphylococcus aureus	1	0	1
	*Staphylococcus aureus *(MRSA)* *	0	1	1
	Staphylococcus epidermidis	0	1	1
	Staphylococcus haemolyticus	0	1	1
	Staphylococcus hominis	1	0	1
	Staphylococcus saprophyticus	1	0	1
Sputum	Acinetobacter baumanii	0	1	1
	Candida albicans	1	2	3
	Candida glabrata	0	1	1
	Candida krusei	1	1	1
	Escherichia coli	1	6	7
	*Enterococcus *spp*. *	0	1	1
	Klebsiella pneumoniae	0	7	7
	Pseudomonas aeruginosa	0	2	2
Bronchial aspirate	Candida albicans	0	1	1
	Escherichia coli	0	1	1
Pus	Staphylococcus aureus	0	1	1
	Citrobacter freundii	0	1	1
	Escherichia coli	0	4	4
	Enterobacter cloacae	0	1	1
	K.pneumoniae	0	1	1
	Proteus mirabilis	0	1	1
	Pseudomonas aeruginosa	0	1	1
Urine	Candida albicans	0	2	2
	Candida tropicalis	0	1	1
	Citrobacter koseri	0	1	1
	Escherichia coli	5	14	19
	*Enterococcus *spp*. *	3	0	3
	Klebsiella pneumoniae	1	5	6
	Proteus mirabilis	0	1	1
	Pseudomonas aeruginosa	1	2	3

The median LOS in the neutropenic patients was 10 days, and that in the non-neutropenic patients was seven days. Patients with male gender (p = 0.011), neutropenia (p = 0.026), and hematological malignancies (p = 0.013) had a longer LOS. Other variables, such as age range (p = 0.169), type of infectious organism (p = 0.281), and outcome (p = 0.447), did not show any significant association with LOS (see Table 5).

**Table 5:** Length of stay (LOS) UTI: urinary tract infection; BSI: blood stream infection; RTI: respiratory tract infection

	Neutropenic episodes		Non-neutropenic episodes	
	Mean	Median	Mean	Median
UTI	7.67	6	7.96	8
BSI	11.27	11	11.67	12
RTI	12	13	7	7
Soft tissue	-	-	9	5.5
BSI + UTI	14	14	16	16

In our study, 12 patients died due to septic shock, resulting in a mortality rate of 12.5%. Neutropenic patients had a significantly higher mortality rate (seven out of 28) as compared to non-neutropenic patients (five out of 63) (OR 3.4, 95% CI 1.178-9.813; p = 0.042). There was no significant association between mortality and the type of malignancy or any age range (p > 0.05). Mortality was exclusively seen in patients with gram-negative infections. All neutropenic mortality had severe neutropenia (ANC <500 cells/mm^3^). It was also observed that five out of the seven neutropenic deaths had profound neutropenia (ANC <100 cells/mm^3^) (see Table 6).

**Table 6:** Summary of the outcome ANC: absolute neutrophil count, UTI: urinary tract infection; BSI: blood stream infection; RTI: respiratory tract infection

		Discharged	Expired	P-value
Gender	Male	44	7	0.699
	Female	40	5	
Malignancy type	Solid	55	5	0.202
	Hematological	29	7	
ANC	Low	20	7	0.042
	Normal	63	5	
Organism	Gram-negative	61	12	-
	Gram-positive	10	0	
	Fungal	11	0	
	Gram-positive and Gram-negative (Two-site infection)	2	0	
Infection type	UTI	31	3	0.68
	BSI	19	5	
	RTI	23	3	
	Soft tissue	9	1	
	BSI+UTI	2	0	

## Discussion

In our study, the majority of infection episodes (70.8%) occurred in the non-neutropenic patients, which is consistent with results from other studies [5,11]. Furthermore, we found that more than two-thirds of neutropenic infection episodes occurred in patients with hematological malignancies, especially in patients with acute myelogenous leukemia (AML) and acute lymphocytic leukemia (ALL).

Among all the episodes of infection, we found that the overall frequency of UTI was the highest and even higher when compared to BSI and respiratory tract infection (RTI), especially in non-neutropenic patients. The early initiation of antibiotic treatment may stop the spread of infection to the bloodstream, which could be one explanation for a higher frequency of UTI than BSI. In this present study, the proportion of patients suffering from BSI was higher in neutropenic patients (55.17%) as compared to non-neutropenic patients (14.49%). This finding corroborates the research results of Chen et al. [12] and Schöning et al. [7]. However, studies by Nørgaard et al. [13] and Velasco et al. [11] showed contrasting results.

In our study, it was found that there was no significant difference in causative microbiology between the neutropenic and non-neutropenic groups. The major causative organism was gram-negative bacteria, followed by gram-positive bacteria and fungal organisms in both neutropenic and non-neutropenic groups. Studies done in the past focusing on BSI patients revealed a preponderance of gram-negative organisms as the major etiological cause [11,13,14]. However, similar results were found in the present study, considering all infection types mentioned in Table 3. We found that *Escherichia* *coli* was the most commonly isolated organism. Other studies showed similar results, where *E. coli* was the most commonly isolated organism in BSI and UTI, suggesting a high burden of *E. coli* infections in cancer patients [11,15-17]. While there was no incidence of polymicrobial infection in our study population, a previous study by Nørgaard et al. [13] in the hematological malignancy population showed that patients with polymicrobial infection had the second highest 30-day mortality. 

The majority of patients recovered from infection, and the overall mortality rate was 12.5%. Results from the study done by Toussaint et al. showed a similar overall mortality rate of 9.5%, but the majority of patients with mortality were non-neutropenic [5]. In our study, mortality was significantly higher in neutropenic patients as compared to non-neutropenic patients (OR 3.4, 95% CI 1.178-9.813; p = 0.042). Similarly, results from Schöning et al. showed an overall mortality rate of only 1.4% in non-neutropenic patients, suggesting some role of neutropenic status on the outcomes. In the present study, mortality occurred exclusively in patients with gram-negative infections. This finding is likely attributed to our small sample size. However, studies done on BSI in neutropenic patients also showed the highest mortality in patients with gram-negative bacteremia [13,18].

We were unable to gather information on comorbidities, Multinational Association for Supportive Care in Cancer (MASCC) score at the time of admission of neutropenic patients, prior corticosteroid medication, or the length of time patients had been receiving cancer treatment because this was a retrospective study that depended on the data found in the patient’s medical records. The possible role of drug-resistant bacteria and indwelling devices on infection incidence and outcome was also not investigated. For a more comprehensive and representative understanding, future studies should aim to validate these findings on a larger scale, involving multiple centers and diverse populations.

## Conclusions

Taken together, our findings suggest that both the neutropenic and non-neutropenic groups did not have much difference in causative microbiology, and both dominated gram-negative organisms. Notably, the microbiological analysis reveals a predominance of gram-negative isolates, with *E.* *coli* emerging as a prominent pathogen in both neutropenic and non-neutropenic patients. Patients with hematological malignancy are more likely to experience neutropenic infectious episodes with a longer stay and poor outcomes. Non-neutropenic infections were seen more in patients with solid malignancy and had better outcomes with a shorter LOS and lower mortality.

This study contributes to existing knowledge by offering comprehensive insights into infections among cancer patients, particularly addressing the comparative characteristics of neutropenic and non-neutropenic episodes. Contrary to some expectations, our findings reveal that neutropenic patients did not exhibit a significantly higher infection frequency compared to their non-neutropenic counterparts. The impact of neutropenia on infection outcomes is explored, underscoring its role as a determinant. The microbiological analysis identifies prevalent gram-negative isolates, informing targeted antimicrobial approaches. The study further reveals distinct infection patterns between neutropenic and non-neutropenic patients, with UTIs being more frequent in the latter and BSIs more prevalent in the former. The investigation associates longer hospital LOS and higher mortality rates with neutropenic episodes, providing clinicians with vital information for intervention strategies and enhancing our understanding of infections in diverse cancer patient populations.

## References

[1] Weycker D, Malin J, Edelsberg J, Glass A, Gokhale M, Oster G (2008). Cost of neutropenic complications of chemotherapy. Ann Oncol.

[2] Maxwell M, Maher K (1992). Chemotherapy-induced myelosuppression. Semin Oncol Nurs.

[3] Sadik M, Ozlem K, Huseyin M, AliAyberk B, Ahmet S, Ozgur O (2014). Attributes of cancer patients admitted to the emergency department in one year. World J Emerg Med.

[4] Bozdemi̇r N, Eray O, Eken C, Şenol Y, Artaç M, Samur M (2009). Demographics, clinical presentations and outcomes of cancer patients admitting to emergency department. Turk J Med Sci.

[5] Toussaint E, Bahel-Ball E, Vekemans M, Georgala A, Al-Hakak L, Paesmans M, Aoun M (2006). Causes of fever in cancer patients (prospective study over 477 episodes). Support Care Cancer.

[6] Bischof JJ, Sylvester PJ, Frey JA (2021). Emergency department disposition of non-neutropenic febrile patients with cancer. J Am Coll Emerg Physicians Open.

[7] Schöning S, Barnbrock A, Bochennek K, Gordon K, Groll AH, Lehrnbecher T (2022). Infections during non-neutropenic episodes in pediatric cancer patients-results from a prospective study in two major large european cancer centers. Antibiotics (Basel).

[8] Feld R, DePauw B, Berman S, Keating A, Ho W (2000). Meropenem versus ceftazidime in the treatment of cancer patients with febrile neutropenia: a randomized, double-blind trial. J Clin Oncol.

[9] Schwartzberg L (2006). Neutropenia: etiology and pathogenesis. Clin Cornerstone.

[10] Punnapuzha S, Edemobi PK, Elmoheen A (2023). Febrile neutropenia. StatPearls [Internet].

[11] Chen CY, Tien FM, Sheng WH (2017). Clinical and microbiological characteristics of bloodstream infections among patients with haematological malignancies with and without neutropenia at a medical centre in northern Taiwan, 2008-2013. Int J Antimicrob Agents.

[12] Nørgaard M, Larsson H, Pedersen G, Schønheyder HC, Sørensen HT (2006). Risk of bacteraemia and mortality in patients with haematological malignancies. Clin Microbiol Infect.

[13] Velasco E, Byington R, Martins CA, Schirmer M, Dias LM, Gonçalves VM (2006). Comparative study of clinical characteristics of neutropenic and non-neutropenic adult cancer patients with bloodstream infections. Eur J Clin Microbiol Infect Dis.

[14] Kanafani ZA, Dakdouki GK, El-Chammas KI, Eid S, Araj GF, Kanj SS (2007). Bloodstream infections in febrile neutropenic patients at a tertiary care center in Lebanon: a view of the past decade. Int J Infect Dis.

[15] Shrestha G, Wei X, Hann K (2021). Bacterial profile and antibiotic resistance among cancer patients with urinary tract infection in a national tertiary cancer hospital of Nepal. Trop Med Infect Dis.

[16] Parikh P, Bhat V (2015). Urinary tract infection in cancer patients in a tertiary cancer setting in India: microbial spectrum and antibiotic susceptibility pattern. Antimicrob Resist Infect Control.

[17] Marín M, Gudiol C, Garcia-Vidal C, Ardanuy C, Carratalà J (2014). Bloodstream infections in patients with solid tumors: epidemiology, antibiotic therapy, and outcomes in 528 episodes in a single cancer center. Medicine (Baltimore).

[18] González-Barca E, Fernández-Sevilla A, Carratalá J, Salar A, Peris J, Grañena A, Gudiol F (1999). Prognostic factors influencing mortality in cancer patients with neutropenia and bacteremia. Eur J Clin Microbiol Infect Dis.

